# Functional Analysis of the Pathogenesis-Related Protein 1 (*CaPR1*) Gene in the Pepper Response to *Chilli veinal mottle virus* (ChiVMV) Infection

**DOI:** 10.3390/v17111456

**Published:** 2025-10-31

**Authors:** Chunzi Huang, Zengjing Zhao, Xing Wu, Hu Zhao, Meng Wang, Zhi He, Zongjun Li, Lihao Wang, Yafei Tang, Risheng Wang, Longfei He, Mingxia Gong

**Affiliations:** 1 Vegetable Research Institute, Guangxi Academy of Agricultural Sciences, Nanning 530007, China; hcz17307806826@163.com (C.H.); nkyzzjing@163.com (Z.Z.); wxscs@gxaas.net (X.W.); zh107100421@126.com (H.Z.); wangmm@gxaas.net (M.W.); rf0073244455@163.com (Z.H.); lizongjun_gxaas@163.com (Z.L.); 2 Guangxi Key Laboratory of Vegetable Breeding and New Technology Development, Nanning 530007, China; 3 College of Agriculture, Guangxi University, Nanning 530004, China; 4 Institute of Vegetables and Flowers, Chinese Academy of Agricultural Sciences, Beijing 100081, China; wanglihao@caas.cn; 5 Plant Protection Research Institute, Guangdong Academy of Agricultural Sciences, Guangzhou 510640, China; yf.tang1314@163.com; 6 Agricultural and Animal Husbandry Industry Development Research Institute, Guangxi University, Nanning 530004, China

**Keywords:** pepper, *PR1* gene, ChiVMV, functional analysis

## Abstract

*Chilli veinal mottle virus* (ChiVMV) causes severe yield losses in pepper across Asia. It is very urgent to study the host plant resistance to control this viral disease. As a type of defense response gene, pathogenesis-related protein 1 (PR1) is a well-established defense marker against fungal/bacterial pathogens, and its role in virus resistance remains unclear. Here, we cloned *CaPR1* from the ChiVMV-highly resistant pepper variety ‘Perennial’. The 477 bp ORF encodes a 17.65 kDa basic protein containing a conserved CAP-PR1 domain. The subcellular localization of *CaPR1* revealed that it was located in the plasma membrane, endoplasmic reticulum (ER), and nucleus. RT-qPCR revealed leaf-predominant expression, with earlier and stronger induction in the highly resistant than the highly susceptible variety after ChiVMV inoculation (6.4-fold at 2 days post-inoculation). The overexpression of *CaPR1* in tobacco significantly increased resistance, reducing disease index by 25% and viral coat protein accumulation. Our findings identified *CaPR1* as a positive regulator of ChiVMV resistance, providing a molecular target for pepper breeding. In addition, exogenous SA treatment increased the resistance of the highly susceptible cultivar ‘Guijiao 12’ to ChiVMV, and 0.25 mM had a greater effect.

## 1. Introduction

Pepper is an economically important crop worldwide. In China, pepper is the largest vegetable crop and has a stable annual production area of more than 2.1 million hm^2^ [1]. In recent years, outbreaks of viral diseases in peppers have become very common, causing severe yield losses and poor fruit quality. More than 30 viruses have been reported for pepper crops in China [2], among which *Chilli veinal mottle virus* (ChiVMV) is considered the most prevalent virus with a broad host range, causing significant yield losses throughout Asia [3,4,5]. ChiVMV, which belongs to the *Potyvirus* genus of the *Potyviridae* family, is transmitted primarily by aphids. Symptoms of ChiVMV infection include dark green mottling with vein banding, necrotic rings or spots, distorted or fallen leaves, fallen flowers, and reduced leaf or fruit size [5,6]. The complete control of plant virus diseases through chemical and physical methods is difficult. Obviously, exploiting host plant resistance is a long-term, eco-friendly, and sustainable strategy to control plant virus diseases.

In plants, the resistance genes (R genes) and the defense response genes are the two major types of genes involved in the resistance response. When a plant is infected by a virus, the R genes can directly or indirectly recognize specific products of the virus (such as coat proteins, movement proteins, replication enzyme proteins, etc.), which trigger a series of downstream defense signal transduction pathways, such as salicylic acid (SA) signal, activate the expression of the downstream defense response genes, like pathogenesis-related protein genes (*PRs*), and ultimately restrict further virus infection [7,8]. To date, nineteen PR families have been categorized based on the major characteristics, such as amino acid sequence similarity, serological relationships, enzyme activities, and pH (acidic or alkaline) [9]. Among them, PR1 was the first protein discovered from *Tobacco mosaic virus* (TMV)-infected tobacco plants [10]. The expression of the *PR1* gene is activated depending on the SA defense signaling pathway and ultimately causes SAR response [11]. Numerous studies over the past 20 years have shown that PR1 has anti-fungal [12], anti-oomycetes [13,14] and anti-bacterial [14,15] activities. For example, overexpression of a pepper-basic PR1 gene, *CABPR1*, in transgenic *Arabidopsis* increased resistance to *Pseudomonas syringae* [16]. However, reports on the function of PR1 in the interaction between plants and viruses are limited. PR1 may also play a key role in plant defense against viruses. For *Capsicum chinense*, the basic PR1 protein isoform was specifically stimulated in the Spanish strain of *Pepper mild mottle virus* (PMMoV-S)-activated *L_3_* gene-dependent resistance [17]. Ren et al. (2020) reported that *CsPR1-1* and *CsPR1-2* were both upregulated in *Cymbidium orchids* after *Cymbidium mosaic virus* (CyMV) infection [18]. Su et al. (2023) reported that soybean *Gm PR1-6* expression was higher in the incompatible combination than in the compatible combination, whereas the resistance to *Soybean mosaic virus* (SMV) decreased after this gene was silenced [19]. However, the function of the *PR1* during the interaction between pepper and ChiVMV remains unclear. Additionally, PR1 genes are seen as a polygenic family within the plant genome; some, but not all, of them are involved in the regulation of disease resistance [18,20]. Hence, it is necessary to clarify the role of the *PR1* gene involved in the defense response to ChiVMV.

During the last twenty years, researchers have performed many studies to identify potyvirus resistance (*pvr*) genes. The majority of *pvr* genes, such as *pvr1*, *pvr2*, *pvr3*, *pvr5*, *pvr6* and *pvr8*, are recessive and associated with the natural mutations of the host factor eIF4E (eukaryotic translational initiation factor 4E) or eIF(iso)4E (the isoform of eIF4E), resulting in passive resistance to potyviruses [21,22]. Among these *pvr* genes, *pvr1*, *pvr2* and *pvr6* confer ChiVMV resistance. According to a previous study, one or more unknown dominant R genes involved in ChiVMV resistance could exist in the highly resistant pepper variety ‘Perennial’ [6]. However, there have been no subsequent reports about the dominant *R* genes, the related signal transduction, and the downstream defense response genes regulated by the *R* genes. Based on our previous transcriptome data from the highly resistant pepper variety ‘Perennial’ and the highly susceptible pepper variety ‘Guijiao 12’, both of which have been stressed by ChiVMV, a downstream defense response gene, *PR1*, with highly differential expression in ‘Perennial’, was screened in response to ChiVMV infection. In this study, the PR1 gene was cloned from ‘Perennial’ via RT-PCR and named *CaPR1*. Based on the nucleotide and the amino acid sequences of *CaPR1*, we analyzed its bioinformatics, subcellular localization, and the expression pattern in various tissues and varieties after ChiVMV inoculation by RT-qPCR. The transformation of the *CaPR1* gene into tobacco was performed using the *Agrobacterium*-mediated method, and the resistance of the transgenic tobacco to ChiVMV was subsequently analyzed. Additionally, we investigated the effect of exogenous salicylic acid (SA) on alleviating ChiVMV infection in ‘Guijiao 12’ (*C. baccatum* L.). The findings of this study will verify the function of PR1 in the interaction between pepper and ChiVMV and lay a good foundation for further exploration of the molecular mechanisms through which *CaPR1* is regulated.

## 2. Materials and Methods

### 2.1. Plant Materials and Viral Inoculation

Two pepper varieties (‘Perennial’ (*Capsicum annuum* L.), highly resistant (HR) to ChiVMV, and ‘Guijiao 12’ (*C. baccatum* L.), highly susceptible (HS) to ChiVMV) were used in this study; their ChiVMV resistance was identified in our previous study [23]. Two tobacco species, *Nicotiana tabaccum* L. cv. Petit Havana SR1 and *N. benthamiana*, were used in this study.

Healthy and ripe seeds of the two varieties were selected for seedling cultivation in a greenhouse in April 2022. Pepper plants with 4–6 real leaves were inoculated by artificial friction with ChiVMV isolated from diseased pepper leaves. The ChiVMV inoculant was prepared as follows: 1 g of diseased leaves infected with only ChiVMV was ground with 100 mL of 0.01 mol∙L^−1^ phosphate buffer (pH 7.2) before filtering. The control plants were also inoculated directly with phosphate buffer. Control and ChiVMV-treated leaf samples were harvested at 2, 4, and 8 days post-inoculation (dpi). The leaves, roots, and stems of normal plants not subjected to any treatment at 2 dpi were also collected. All the samples were quickly frozen in liquid nitrogen and preserved at −80 °C until subsequent analyses were conducted. Every treatment was performed thrice.

### 2.2. RNA Isolation and cDNA Synthesis

The plant tissues were ground into powder in liquid nitrogen using mortar and pestle. RNA was isolated using an RNA Pure Plant Kit (Kangwei, CW0588S, Beijing, China). The RNA integrity was determined using a 1.5% agarose gel; thereafter, the RNA quality and content were detected with an ultramicrospectrophotometer. The RNA samples were then reverse-transcribed into cDNA with a HiFiScript cDNA Synthesis Kit (Kangwei, CW2569M, Beijing, China) in a 20 µL reaction mixture.

### 2.3. Gene Cloning and Bioinformatics Analysis

The specific primers *CaPR1*-F/*CaPR1*-R were designed according to the *CaPR1* gene sequence via the CE design tool listed in Table 1. The cDNA of the highly resistant variety was used as a template to amplify the expected fragment via polymerase chain reaction (PCR). The expected fragment was recovered and connected to T-Vector pMD™ 19, transferred into *E. coli* DH5α, screened for blue and white spots, and detected in bacterial solution via PCR. Finally, three positive clones were sent to Sangong Biological Company for sequencing (Sangon Biotech Co., Ltd., Shanghai, China). The positive clones were picked out, cultured, and used to extract vector plasmids to prepare a DNA template for subsequent experiments.

ORF finder was used to identify the ORF and deduce the protein sequence, and BLASTP on the NCBI website was used to analyze conserved domains. Diverse physicochemical indicators for the CaPR1 protein were computed with ProtParam (https://web.expasy.org/protparam/, accessed on 8 April 2022). The transmembrane helices and the secreted signal peptide in the protein were estimated using the DeepTMHMM (https://dtu.biolib.com/DeepTMHMM, accessed on 8 April 2022 ) tool and the SignalP 6.0 tool (https://services.healthtech.dtu.dk/, accessed on 3 January 2022), respectively. Protein subcellular localization, three-dimensional structure, and generic phosphorylation sites were predicted using the WoLF POSRTII tool (https://www.genscript.com/wolf-psort.html, accessed on 24 November 2022), the SWISS-MODEL tool (https://swissmodel.expasy.org/, accessed on 2 July 2018), and the NetPhos-3.1 (https://services.healthtech.dtu.dk, accessed on 1 January 2024/), respectively. Other plant PR1 sequences were retrieved from the GenBank database. MEGA 11.0 was used for phylogenetic analyses of the amino acids (AAs) using the neighbor-joining approach. The tree reliability was assessed using 1000 bootstrap replicates.

### 2.4. CaPR1 Subcellular Localization

The stop codons of the open reading frame (ORF) of *CaPR1* were removed, and a pair of pBI121-*Xho*I-F and pBI121-*Sal*I-R primers (Table 1) with cleavage sites *Xho*I and *Sal*I, respectively, was designed, with 15–20 bp homologous arm sequences of the pBI121-GFP vector. The ORF of *CaPR1*, with the stop codon removed, was amplified via specific primers to recombine with the vector. The pBI121-GFP vector was subsequently subjected to *Xho*I and *Sal*I enzyme digestion before ligation with target PCR products using the ClonExpress II One Step Cloning Kit (Vazyme Biotech Co., Ltd., Nanjing, China). Next, the freeze–thaw technique was used to transfect a recombinant vector or empty control vector into *Agrobacterium tumefaciens* strain GV3101. *Agrobacterium* cells containing the pBI121-GFP or *CaPR1*-pBI121-GFP vector were subsequently inoculated in LB medium supplemented with 50 μg∙mL^−1^ kanamycin (Kan) or 20 μg∙mL^−1^ rifampicin (Rif). Later, we detected the *Agrobacterium* mixture, diluted it to OD_600_ = 0.2–1.0, and then infiltrated it into the third and fourth leaves from the top down of the *N. benthamiana* seedlings at the eight-leaf stage. These seedlings were subsequently maintained in the dark for one night and grown for two days in the greenhouse. The infiltrated leaves were collected and visualized using a confocal laser scanning microscope (UltraVIEW VOX, PerkinElmer, Waltham, MA, USA) at capture and excitation wavelengths of 448–508 nm and 488 nm, respectively.

### 2.5. Overexpression Vector Establishment and Genetic Transformation of Tobacco

According to the entire coding sequence (CDS) in the *CaPR1* gene, pCAMBI1301-*Bgl*I-F and pCAMBI1301-*Bst*EII-R primers (Table 1) with restriction sites *Bgl*I and *Bst*EII, respectively, and both with 15–20 bp homologous arm sequences of the pCAMBI1301 vector were designed. The entire CDS of *CaPR1* was amplified using specific primers, after which the target product was gel-purified. The pCAMBI1301 vector was double-digested with *Bgl*I and *Bst*EII enzymes before ligation with a purified product using the ClonExpress II One Step Cloning Kit (Vazyme Biotech Co., Ltd., Nanjing, China) to obtain the overexpression recombinant vector pCAMBI1301-*CaPR1*. After validation through double-enzyme digestion and sequencing, this recombinant vector was transformed into *A. tumefaciens* strain GV3101 by the freeze–thaw technique. *Agrobacterium* cells were inoculated in LB medium supplemented with 50 μg∙mL^−1^ Kan or 20 μg∙mL^−1^ Rif together with 25 μg∙mL^−1^ hygromycin (Hyg). The positive *Agrobacterium* solutions were measured and diluted to OD_600_ = 0.5–0.6 for the genetic transformation of SR1 tobacco via a leaf disc approach. Later, the Kan selective medium was used to screen out the transgenic plants, and the target gene *CaPR1* was amplified by PCR.

### 2.6. Gene Expression Analysis

To investigate the expression of the *CaPR1* gene in diverse pepper tissues, the cDNA of the root, stem, and leaf samples of two pepper varieties, ‘Perennial’ and ‘Guijiao 12’, was used as the template. To verify that the *CaPR1* gene responds to viral stress, the expression of *CaPR1* was analyzed using leaf cDNA samples from ‘Perennial’ and ‘Guijiao 12’ plants inoculated with ChiVMV at 2, 4, and 8 dpi. To analyze *CaPR1* expression in T_2_ transgenic tobacco plants, cDNA samples from T_2_ transgenic lines and wild-type (WT) tobacco were used as templates for RT-qPCR. Moreover, a relative quantitative analysis of the ChiVMV coat protein-encoding gene *cp* in T_2_ transgenic lines and WT tobacco was performed. The RT-qPCR assays were performed with a Roche LightCycler 480 System (Roche, Basel, Switzerland) using SYBR GoTaq^®^ qPCR Master Mix (Promega, Beijing, China). PCR for each sample was conducted three times with 0.5 µL of each primer (10 µM), 1 µL of cDNA, and 5 µL of SYBR qPCR master mix in a final volume of 10 µL. The cycling conditions were as follows: 5 min of denaturation at 95 °C, followed by 40 cycles of 95 °C for 15 s and 60 °C for 30 s. Pepper *CaActin* (AY572427.1) served as a normalized endogenous reference gene. The 2^−△△Ct^ approach was used to determine gene expression. Each assay was performed with three biological replicates and three technical replicates. The primers used for RT-qPCR are listed in Table 1.

### 2.7. Assessment of ChiVMV Resistance in Transgenic Tobacco

The WT tobacco and T_2_ transgenic tobacco plants at the four-leaf stage were inoculated with ChiVMV using the same method as described above, with three independent biological replicates. Each biological replicate included thirty plants. The phenotypic characteristics of these plants in response to ChiVMV infection were observed at 15 dpi. Moreover, we assessed plant disease-associated parameters by the method described in our previous study [23] at 30 dpi. The disease index (DI) was determined as follows to assess ChiVMV resistance in plants. Disease resistance was divided into six levels according to the DI, as shown in Table 2.

$$Disease\;index\left(DI\right)=\frac{\sum (Representative\;value\;of\;incidence\;level\times number\;of\;diseased\;plants\;of\;this\;level)}{Total\;number\;of\;investigated\;plants\times representative\;value\;of\;the\;highest\;incidence\;level}\times 100$$

### 2.8. Treatment with Salicylic Acid

After inoculation with ChiVMV for 20 d, the highly susceptible ‘Guijiao 12’ plants were sprayed with salicylic acid (SA) at three concentrations (0.1, 0.25, and 0.5 mM), and water was used as a control. Each treatment was repeated three times, and each replicate included 30 plants. Leaves from the five random plants of each replicate were collected at 4 and 15 days post-spraying (dps) and used for the detection of the expression level of the *CaPR1* and *cp* genes, respectively. The disease symptoms of other plants were observed to calculate the DI at 15 dps.

### 2.9. Statistical Analysis

The results are presented as the mean ± standard error (SE) of three replicates. The data were analyzed by one-way ANOVA followed by Tukey’s HSD test using SPSS 19.0 (IBM, New York, NY, USA); *p* < 0.05 and *p* < 0.01 indicated statistical and extreme statistical significance, respectively.

## 3. Results

### 3.1. Cloning and Bioinformatic Analysis of the CaPR1 Gene

**Figure 1:**
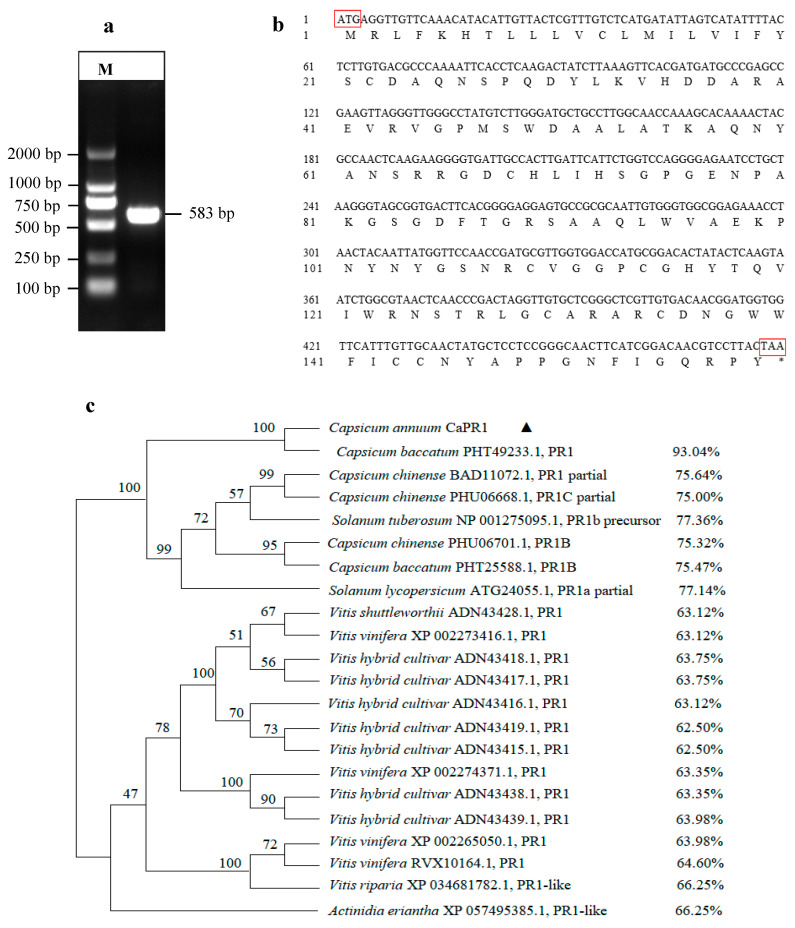
(**a**) *CaPR1* gene cloning and sequences, M: DNA Marker DL2000. (**b**) *CaPR1* ORF sequence and its deduced amino acid sequence. (**c**) Phylogenetic tree of homologous PR1 protein of different species; the percentage following each entry indicates the sequence identity with CaPR1.

The *CaPR1* gene was amplified through PCR with ‘Perennial’ cDNA as the template, and a fragment with an expected size of 583 bp was obtained by agarose gel electrophoresis (Figure 1a). The nucleotide sequence was obtained through gel recovery, cloning, and sequencing. The ORF in the *CaPR1* gene is 477 bp long and encodes 158 amino acids (Figure 1b). The amino acid sequence alignment of pepper CaPR1 with other plant PR1 proteins revealed that CaPR1 amino acids shared the highest sequence identity (93.04%) with the PR1 of *C. baccatum*. Phylogenetic tree analysis revealed that the amino acid sequence of CaPR1 was the most closely related to PR1 of *C. baccatum*, followed by PR1, PR1C, and PR1B of *C. Chinese*, the PR1b precursor of potato and PR1a of tomato. However, it had a distant relationship with PR1 in grape and kiwi (Figure 1c).

**Figure 2:**
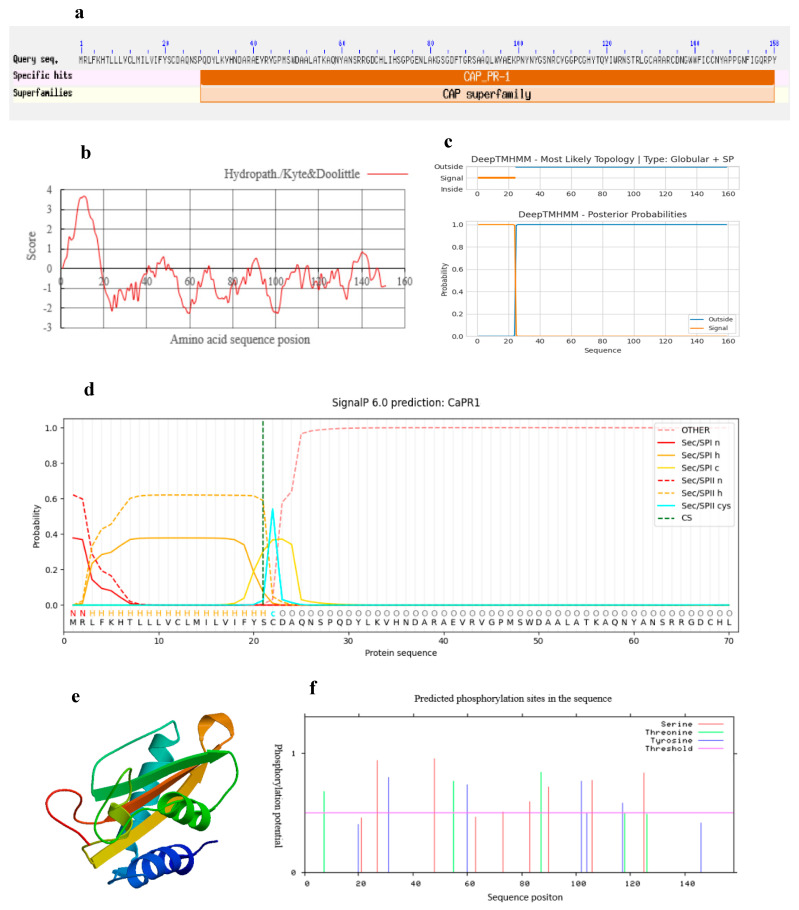
Bioinformatics analysis of the CaPR1 protein. (**a**) Conserved domain prediction. (**b**) CaPR1 protein affinity/hydrophobicity prediction. (**c**) Transmembrane structure prediction. (**d**) Signal peptide prediction (Sec/SPII indicates the lipoprotein signal peptide). (**e**) Protein three-dimensional structure prediction. (**f**) Phosphorylation site prediction.

The molecular weight of the CaPR1 protein is 17.65 kDa, and its theoretical isoelectric point (p*I*) is 8.91. According to the SMART and NCBI conserved domain databases, the deduced CaPR1 protein contains one conserved CAP-PR1 domain and one CAP domain located from amino acids 28–158, belonging to the PR1 family of the CAP superfamily (Figure 2a). The CaPR1 sequence had more hydrophilic (negative value) than hydrophobic (positive value) amino acids, the total average hydrophilic index was −0.392, and the instability coefficient was 38.19%, suggesting that CaPR1 is stable and hydrophilic (Figure 2b). Additionally, the deduced CaPR1 protein did not contain a transmembrane helical structure, indicating that it is not a transmembrane protein (Figure 2c). SignalP analysis revealed a 24 AA lipoprotein signal peptide (SP) region of the CaPR1 protein at its N-terminus, and the predicted cleavage site was between AA 21 and 22 (Figure 2d). The three-dimensional structure of the CaPR1 protein was constructed and is shown in Figure 2e (GMQE value 0.77, Seq identity 78.20%), which contained four *α*-helices and four *β*-folds, several random coils, and extended fragments, forming a stable *α-β-α* sandwich structure shared by the PR1 proteins. Protein modification analysis revealed nine serine (Ser), five threonine (Thr), and seven tyrosine (Tyr) phosphorylation sites and no O-glycosylation site or N-glycosylation site in the CaPR1 protein sequence (Figure 2f). Subcellular localization of the CaPR1 protein was conducted with WoLF PSORT, which predicted that CaPR1 could be located in the extracellular matrix, chloroplast, and nucleus.

### 3.2. CaPR1 Expression Analysis

**Figure 3:**
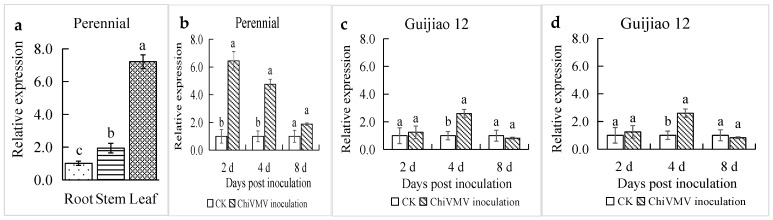
(**a**,**b**) Expression level of *CaPR1* in different organs of the two pepper varieties. (**c**,**d**) Expression level of *CaPR1* in two pepper varieties after inoculation with ChiVMV. Different lowercase letters above the bars indicate a significant difference (*p* < 0.05).

The expression of *CaPR1* was detected in different organs, such as in the roots, stems, and leaves of both pepper varieties, through RT-qPCR. Its expression differed in different organs and was sorted by the magnitude of expression level as follows: leaves > stems > roots. *CaPR1* expression in the leaves was 7.2-fold and 4.8-fold greater than that in the roots of ‘Perennial’ (HR) and ‘Guijiao 12’ (HS), respectively (Figure 3a,b). To further investigate the response of *CaPR1* to ChiVMV infection, its relative expression in the leaves of two pepper varieties under uninoculated and inoculated ChiVMV treatments was determined by qRT-PCR. Compared with those in the uninoculated control, the expression levels of *CaPR1* in ‘Perennial’ (HR) increased markedly at 2 and 4 dpi, peaking as early as 2 dpi, and were 6.4-fold higher at 2 dpi (Figure 3c). In ‘Guijiao 12’ (HS), ChiVMV infection increased *CaPR1* gene expression to increase by 2.5-fold at 4 dpi compared with that in the uninoculated plants (Figure 3d). Additionally, *CaPR1* expression in highly resistant ‘Perennial’ plants was higher than that in highly susceptible ‘Guijiao 12’ plants on the same day after ChiVMV inoculation.

### 3.3. Subcellular Localization Analysis of CaPR1

**Figure 4:**
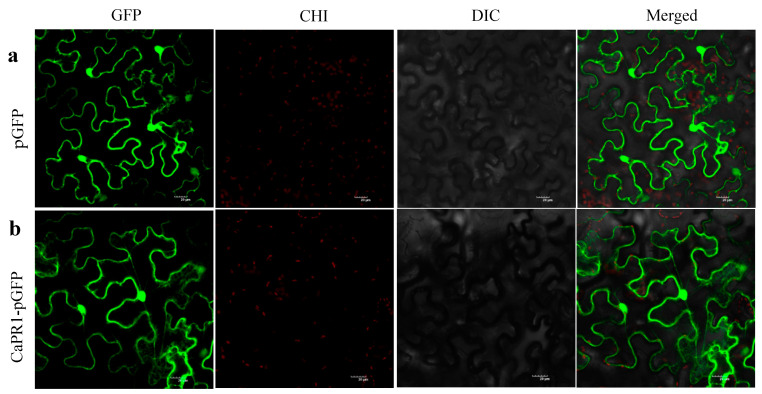
Transient expression of *CaPR1* in tobacco epidermal cells. (**a**) pGFP: GFP protein in the empty vector. (**b**) CaPR1-pGFP: fusion protein of CaPR1 and GFP in recombinant vector. GFP: Green fluorescence field; CHI: Chloroplast autofluorescence field; DIC: Bright field; Merged: Superposition field. Scale bar = 20 µm.

Subcellular localization analysis of CaPR1 was performed by conducting a tobacco transient expression assay. The C-terminus of the CaPR1 protein was integrated with the N-terminus of the fluorescent protein to transform tobacco epidermal cells. The fluorescence signal of the fusion protein was observed under a confocal laser microscope. Confocal microscopy revealed the distribution of the control pGFP signal in the plasma membrane, endoplasmic reticulum (ER), and nucleus (Figure 4a). CaPR1-GFP signals were visualized in the plasma membrane, ER, and nucleus (Figure 4b). Based on these results, we concluded that the CaPR1 protein was localized to the plasma membrane, ER, and nucleus.

### 3.4. Overexpression of CaPR1 Enhanced Resistance to ChiVMV

**Figure 5:**
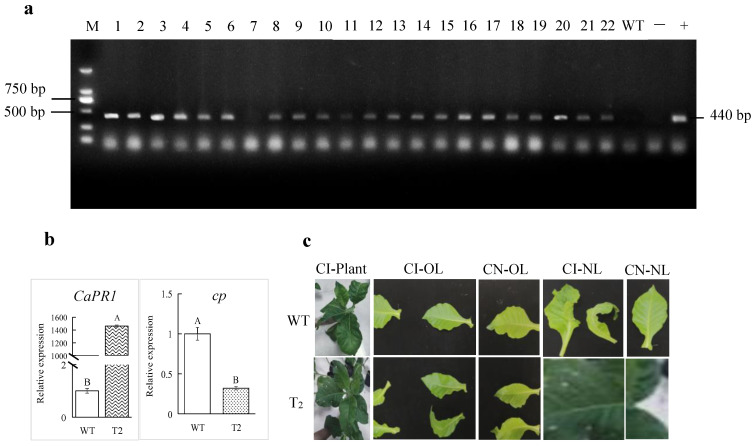
(**a**) PCR identification of T_0_ transgenic tobacco lines, M: DNA marker DL2000; 1–22: T_0_ transgenic tobacco lines; WT: wild-type tobacco (the same below); −: negative control (water); +: positive control (pCAMBI1301-CaPR1 recombinant plasmid). (**b**) Expression level of the *CaPR1* and *cp* genes in the leaves of T_2_ transgenic and WT tobacco plants at 15 dpi. Different capital letters above the bars indicate highly significant differences (*p* < 0.05). (**c**) Phenotypes of tobacco plants at 15 dpi. T_2_: T_2_ transgenic tobacco; CI: ChiVMV inoculation; CN: ChiVMV uninoculation; OL: Old leaves; NL: New leaves.

The gene overexpression vector pCAMBI1301-*CaPR1* was constructed and subsequently used to generate the transgenic tobacco plants through the *Agrobacterium*-mediated technique. The genomic DNA of *CaPR1* transgenic and wild-type (WT) tobacco plants was extracted and used as a template for PCR detection via the specific primers SR2-CaPR1-F and SR2-CaPR1-R. The PCR results revealed that 21 of the 22 T_0_ transgenic tobacco lines amplified a 440 bp target fragment, which was consistent with the positive control (Figure 5a). The positive plants were propagated vegetatively in MS media and then transferred to soil for T_1_ seeds. Some of the T_1_ lines with high positive rates were selected and propagated for T_2_ seeds. T_2_ transgenic tobacco plants were obtained for subsequent functional identification.

Compared with WT plants under normal conditions, in transgenic T_2_ tobacco plants, the *CaPR1* expression level increased over 1400-fold, suggesting that *CaPR1* was overexpressed in those plants (Figure 5b). The phenotypes of the transgenic T_2_ tobacco-overexpressing plants were observed in response to ChiVMV infection. The old and new leaves of the WT plants were mosaic, with severe shrinkage, deformity, and the formation of dead spots at 15 dpi. However, the leaves of T_2_ tobacco plants were less severely affected than those of the WT plants (Figure 5c). Furthermore, the DI of the tobacco plants inoculated with ChiVMV was investigated at 15 dpi. The transgenic lines presented a 25% reduction in the disease index (37.78% vs. WT 50.37%; *p* < 0.01, Table 3) and 3.2-fold lower viral *cp* expression (Figure 5b), indicating that their resistance to ChiVMV was significantly increased and that *CaPR1* had a positive regulatory effect on ChiVMV infection.

### 3.5. SA Treatment Enhanced Resistance to ChiVMV

**Figure 6:**
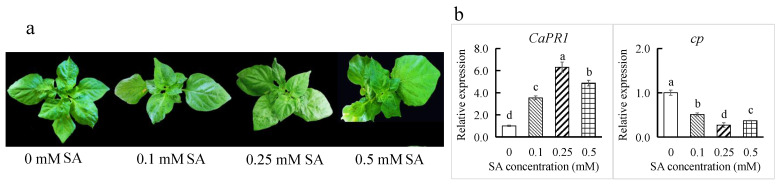
Effect of SA spraying on (**a**) the phenotypes and (**b**) the relative expression of the *CaPR1* at 4 dps and the *cp* gene at 15 dps in ‘Guijiao 12’ plants infected with ChiVMV. Different lowercase letters above the bars indicate a significant difference (*p* < 0.05).

To increase pepper resistance to ChiVMV, exogenous SA treatment was applied to the highly susceptible ‘Guijiao 12’ plants infected with ChiVMV. At 15 dps, the two unfolded new leaves of the plant top showed less severe wrinkling and deformation (Figure 6a), and the whole plants had significantly lower levels of DI (shown in Table 4) in the SA treatment groups than in the control. The DI of ‘Guijiao 12’ was the lowest in the 0.25 mM SA treatment, followed by the 0.5 and 0.1 mM SA treatments. As the SA concentration increased from 0 to 0.25 mM, the *CaPR1* expression increased significantly, whereas the *cp* expression decreased significantly (Figure 6b). These results indicated that SA treatment could stimulate the *CaPR1* expression and suppress the proliferation of ChiVMV. However, compared with that in the 0.25 mM SA treatment, *CaPR1* expression was significantly lower, and the *cp* expression was significantly greater in the 0.5 mM SA treatment. These results proved that 0.5 mM SA was beyond the appropriate concentration range. Hence, these results showed that among the different concentrations tested, 0.25 mM SA was more effective at increasing the resistance of the highly susceptible cultivar ‘Guijiao 12’ to ChiVMV.

## 4. Discussion

Pepper is an economically important crop that faces tremendous losses after being infected by ChiVMV. The study of the host plant resistance to control this viral disease is very urgent. As a defense response gene, the *PR1* gene is a reliable indicator of SAR activation in various plant species [11]. In this study, a *PR1* gene named *CaPR1* from the highly resistant pepper variety ‘Perennial’ was cloned. It encoded 158 amino acids and presented the highest similarity (93.04%) with the PR1 protein of *C. baccatum.* Bioinformatics analysis of the CaPR1 protein was conducted, and the main results were as follows. First, the CaPR1 protein, with a p*I* value of 8.91, was basic, which was consistent with the AvPR1 protein of oats reported in another study [24]. The p*I* value of PR1 may be related to its subcellular localization. Generally, the PR1 proteins distributed in the intercellular space are mostly acidic proteins with p*I* values less than seven, whereas those present in vacuoles are mostly basic proteins with p*I* values greater than seven [25,26]. Second, the CaPR1 protein was found to be located in the plasma membrane, ER, and nucleus through a tobacco transient expression assay. Its location in the plasma membrane and ER has been reported in other studies; however, its location in the nucleus is rare, suggesting its function [9,11,27,28]. Previous studies have reported that several infection processes of *Potyvirus,* such as *Tobacco etch virus* (TEV) and *Potato virus Y* (PVY), are associated with the plant host membranes, the ER and the nucleus [29,30]. The same is true for ChiVMV, another member of *Potyvirus.* Thus, with respect to the localization of CaPR1 in the plasma membrane, the ER, and the nucleus, it aligns with potential potyviral replication sites. Third, the deduced CaPR1 protein contained an SP at its N-terminus. SP usually transiently extends to the amino terminus of proteins and can guide proteins in subcellular organelles that have diverse membranous structures [9,31]. Hence, CaPR1 protein might be guided onto these organelles via the N-terminal SP to execute its biological activities.

The PR1 family is a member of the CAP superfamily, which consists of cysteine-rich secretory protein (CRISP) from humans, insect-derived antigen 5 (Ag5), and PR1 from plants [32,33]. Most PR1 family members contain only a CAP domain apart from relatively short C- and N-terminal extensions, indicating that the CAP domain determines their function in plant pathogen defense [34]. Here, the *CaPR1* protein also contained a conserved CAP-PR1 domain and a stable *α-β-α* sandwich structure, which is consistent with the previous research conclusions [9,11,14]. Previous studies have shown that the CAP domain has an antimicrobial effect because of its caveolin-binding motif (CBM) with a sterol-binding function [14]. This means that CBM may be involved in binding to sterols in the membrane of host plants or pathogens and may directly attack or inhibit pathogens when the host is infected [11,14,35,36]. Caveolin-1 is the most important structural protein of caveolae, a type of membrane invagination widely known for its role in endocytosis and subsequent cytoplasmic transportation during virus infection [37]. In plants, viruses enter host cells via non-endocytic pathways, such as mechanical inoculation or insect-mediated infection. The subsequent post-entry events, including replication, assembly, and egress of many viruses, are dependent on caveolae-mediated endocytosis [37,38,39,40]. Sterols are a major class of lipids found in the membranes of all eukaryotes. The binding of sterols by CAP proteins is not specific to a particular type of sterol, as these proteins can bind the plant sitosterol or the fungal ergosterol [34]. Sequestering sterols from the membrane by PR1 proteins may increase the membrane fluidity and induce ROS production, according to a previous study [41]. In the interaction between viruses and plant hosts, abundant H_2_O_2_ is frequently produced in the incompatible combination, whereas less or no obvious H_2_O_2_ was observed in the compatible combination [42,43]. As a second intracellular messenger, H_2_O_2_ is involved in regulating the accumulation of callose deposition on plasmodesmata, thereby inhibiting the spread of viruses, such as TMV and SMV [42,44]. Hence, it was speculated that the predicted CAP domain and the localization of the CaPR1 protein suggest its potential involvement in membrane-associated defense during the interaction between ChiVMV and pepper.

In pepper, the expression levels of *CaPR1* in different tissues were different, and the highest level was in leaf tissues, indicating that this gene likely performs its function mainly in leaf tissues. In tomato, *SlPR1* was expressed at the greatest levels in stem tissues [15]. In potato, 22 *PR1* genes presented the highest levels in root tissues [45]. Here, our study also revealed that the *CaPR1* gene appeared to be inducible. This gene was upregulated in two pepper varieties subjected to ChiVMV infection, similar to the result of a previous study that the *CaBPR1* gene can be induced by TMV in pepper plants [13]. Ren et al. (2020) reported that *CsPR1-1* and *CsPR1-2* were induced to express in *Cymbidium orchids* after CyMV infection [18]. In soybean, *GmPR1-6* expression was significantly upregulated after SMV inoculation [19]. Additionally, the *CaPR1* expression in the highly resistant variety peaked earlier (at 2 dpi) and was significantly greater on the same day after ChiVMV inoculation than that in the highly susceptible variety. These results revealed that the defense response of the *CaPR1* gene against ChiVMV infection was greater in the highly resistant variety than in the highly susceptible variety, which is consistent with the findings of a previous study confirming that differences in susceptibility and resistance are related to differences in the timing and magnitude of the induced response [17]. Hence, the *PR1* expression level is often used as an indicator of the strength of the defense response in plants. Notably, virus infections often have early events, and the induction of PR1 may occur very early. Hence, the peak level of the *CaPR1* expression in the highly resistant variety may have occurred before 2 dpi.

Compared with that of the WT tobacco plants in this study, the resistance to ChiVMV of the *CaPR1* transgenic lines was significantly greater, verifying that *CaPR1* has an anti-ChiVMV role. Su et al. (2023) reported that the resistance of soybean to SMV decreased after the gene *Gm PR1-6* was silenced [19]. In plants, SA does not inhibit the pathogen growth directly but rather induces systemic acquired resistance (SAR), which is correlated with the expression of PR proteins and defense-related enzymes in defense response to viruses, for example, *Mungbean yellow mosaic virus* (MYMV) in *Vigna mungo* and *Tomato yellow leaf curl virus* (TYLCV) in tomato [46,47,48]. Our results showed that the exogenous application of SA sharply induced *CaPR1* accumulation and alleviated the disease symptoms in new leaves of pepper plants infected with ChiVMV. In *Capsicum annuum* L., among different concentrations (50, 100, and 150 ppm), 100 ppm SA was proven to be effective against ChiVMV [49]. In tomatoes, 2 mM SA could increase resistance to TYLCV by inducing the expression of PR genes such as *PR1* and altering the activity of resistance-related enzymes [47]. These studies also revealed that the optimum concentration of SA for various plants to induce a defense response to viral diseases is different [50]. Here, in *C. baccatum* L., 0.25 mM SA was more effective than 0.5 mM SA in improving resistance to ChiVMV, and the expression level of *CaPR1* was greater in 0.25 mM SA than that in 0.5 mM SA. How can this result be explained? This finding might be related to the regulatory molecular mechanisms of the *PR1* gene in plant defense, which have been researched broadly. *NPR1* (nonexpressor of pathogenesis-related gene 1) can positively regulate SA signaling in plants, which acts as a transcriptional cofactor and works together with other transcription factors such as TGA, bZIP, and WRKY to modulate the activity of the *PR1* gene promoter, ultimately regulating *PR1* gene expression [51,52,53,54,55,56]. NPR1 paralogs NPR3 and NPR4 are SA receptors that bind SA with different affinities, which mediate NPR1 degradation in an SA-regulated manner [57]. When the SA level is very high, NPR3 binds NPR1 and degrades the NPR1 in the nucleus; when SA level is modest, it represses the interaction between NPR4 and NPR1 and is not sufficient to promote the interaction between NPR3 and NPR1. Under these conditions, the NPR1 remains at a relatively high level to induce the expression of the downstream genes such as PR1 [57]. Hence, we speculated that 0.5 mM SA might be too high for *C. baccatum* L. to maintain NPR1 at a relatively high level and induce the expression of *CaPR1*, leading to a poor effect on enhancing the resistance of *C. baccatum* L. to ChiVMV. The real mechanisms involved in regulating *PR1* gene expression in defense response to ChiVMV need further investigation.

## 5. Conclusions

To summarize, we cloned a *PR1* gene in pepper (named *CaPR1*) and conducted bioinformatics characterization and phylogenetic analysis. Subcellular localization analysis revealed that CaPR1 was localized in the plasma membrane, ER, and nucleus. Among the three pepper tissues, the *CaPR1* gene exhibited the highest expression in leaves. ChiVMV infection could induce *CaPR1* expression in the leaves of both pepper varieties. *CaPR1* expression was considerably higher in highly resistant ‘Perennial’ than in highly susceptible ‘Guijiao 12’ after ChiVMV inoculation. The overexpression of *CaPR1* gene could significantly enhance the resistance of transgenic tobacco plants to ChiVMV. These results revealed the positive role of the *CaPR1* gene in pepper defense response to ChiVMV infection. Additionally, exogenous SA treatment could induce the *CaPR1* expression and ultimately enhance the plant’s resistance to ChiVMV.

## Figures and Tables

**Table 1:** Primer information used in this study.

Use	Primer Name	Primer Sequence (5′-3′)	Product Length (bp)
Gene cloning	*CaPR1*-F	CTCCACTAGAACTAAAACAC	583
	*CaPR1*-R	ATTATCAACCCCCTTAGCTT	
RT-qPCR	*Actin*-QF	TTGGGATGATATGGAGAAGATATGGCATC	147
	*Actin*-QR	AACGTCTCAAACATAATCTGGGTCATCT	
	*CaPR1*-QF	GAGCCGAAGTTAGGGTTGGG	122
	*CaPR1*-QR	ACCGCTACCCTTAGCAGGAT	
	ChiVMV-*cp*-QF	GGATGTTCGGATTGGACGGT	97
	ChiVMV-*cp*-QR	CCCAGCAGGTTGTGCATATTTC	
ORF amplification (without TC)	pBI121-*XhoI*-F	ctcgagATGAGGTTGTTCAAACATACATTGTTAC	474
	pBI121-*SalI*-R	gtcgacGTAAGGACGTTGTCCGATGAAGT	
ORF amplification (with TC)	pCAMBI130-*BglI*-F	agatctATGAGGTTGTTCAAACATACATTGTTAC	477
	pCAMBI1301-*BstEII*-R	ggtcaccTTAGTAAGGACGTTGTCCGATGAA	
Amplification of target gene fragment	SR1-*CaPR1*-F	TGACGCACAATCCCACTATC	440
	SR1-*CaPR1*-R	ATGGTCCACCAACGCATC	

Note: TC in the table means “termination codon”.

**Table 2:** Evaluation criteria for tobacco resistance to ChiVMV disease.

Disease Index (DI)	**Resistance Evaluation**
DI = 0	Immune (I)
0 < DI < 10	Highly resistant (HR)
10 ≤ DI < 20	Resistant (R)
20 ≤ DI < 40	Moderately resistant (MR)
40 ≤ DI < 60	Susceptible (S)
60 ≤ DI	Highly susceptible (HS)

**Table 3:** Tobacco disease index after ChiVMV inoculation *.

Material Type	DI (%)	Disease Resistance Level
WT	50.37 ± 1.28 A	Susceptible (S)
T_2_	37.78 ± 2.22 B	Moderately resistant (MR)

*: Different capital letters following data in the same column indicate an extremely significant difference. (*p* < 0.01).

**Table 4:** Effect of SA spraying on the DI of Guijiao 12 infected with ChiVMV *.

SA Concentration (mM)	DI (%)
0 (CK)	83.46 ± 2.27 Aa
0.1	76.21 ± 2.36 Ab
0.25	59.84 ± 3.13 Bc
0.5	66.08 ± 2.51 Bc

*: Different capital or lowercase letters following data in the same column indicate an extremely significant (*p* < 0.01) or significant (*p* < 0.05) difference, respectively.

## Data Availability

Data is contained within the article. Further inquiries can be directed to the corresponding authors.
